# Exploration of the feasibility of the tunneling technique for colonic lesions originating from the muscularis propria

**DOI:** 10.1055/a-2466-9794

**Published:** 2024-12-03

**Authors:** Bin-Yang Luo, Xia-Fei Gu, Zhu Wang

**Affiliations:** 1 34753 Department of Gastroenterology and Hepatology, West China Hospital of Sichuan University, Chengdu, China; 2 34753 Department of Pathology, West China Hospital of Sichuan University, Chengdu, China

Submucosal tunneling endoscopic resection (STER) was developed for treatment of upper gastrointestinal submucosal tumors originating from the muscularis propria, with its safety well-established through extensive clinical evidence. However, reports of its application in the resection of colonic lesions have been scarce. The thinner colonic wall, combined with the presence of colonic folds, substantially increases the complexity of the tunneling technique. In a recent case, we successfully employed the STER technique to treat a mass originating from the muscularis propria in the colon, demonstrating its feasibility in this context ( Video 1 ).

Submucosal tunneling endoscopic resection for a colonic lesion originating from the muscularis propria.Video 1

A 68-year-old female patient underwent colonoscopy, which revealed a submucosal tumor located in the transverse colon. Endoscopic ultrasound (EUS) revealed a round mass originating from the muscle layer, characterized by mild hypoechogenicity. ( Fig. 1 ). The mucosa was incised on the fluid cushion using a DualKnife (Olympus), allowing access to the submucosal layer. A tunnel was created using both the DualKnife and an ITknife (Olympus); meticulous dissection across the colonic folds was required to avoid mucosal injury. Upon visualization of the tumor, it was carefully separated from the surrounding tissue between the mucosa and the muscularis propria. Following tumor excision, the serosal membrane and surrounding adipose tissue were exposed, with no evidence of intraoperative pneumoperitoneum. The tunnel was preserved postoperatively, and the entry site was closed using endoscopic clips ( Fig. 2 ). Pathological examination confirmed the diagnosis of a schwannoma measuring approximately 2.2 cm × 2 cm ( Fig. 3 ).

**Fig. 1:**
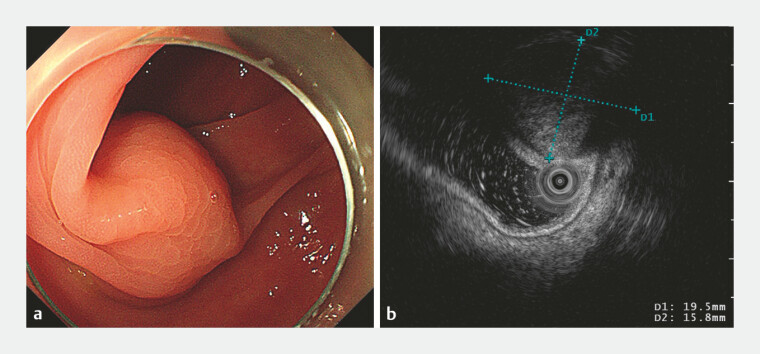
**a** A submucosal tumor with a smooth surface located in the transverse colon. **b** Endoscopic ultrasound (EUS) revealed a 19-mm × 16-mm mildly hypoechoic mass originating from the muscularis propria.

**Fig. 2:**
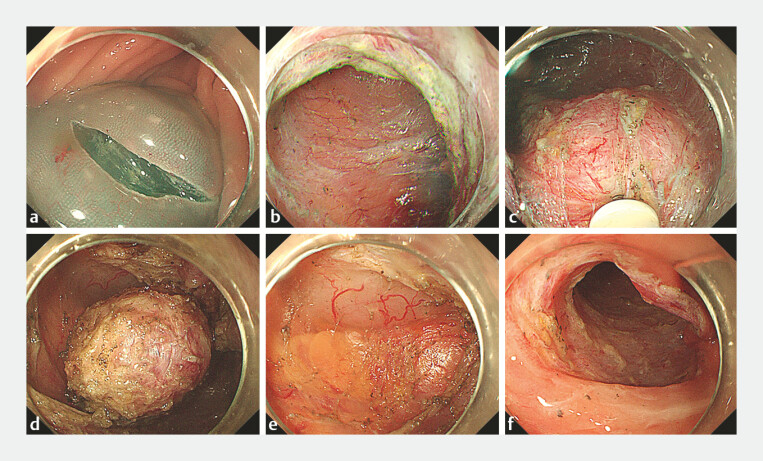
**a** A transverse incision at the anal side of the lesion. **b** Creation of the tunnel. **c** Visualization of the tumor. **d** The tumor was separated from surrounding tissues. **e** Serous membrane and surrounding adipose tissue were visible at the resected site. **f** Entrance to preserved tunnel. This was closed with clips.

**Fig. 3:**
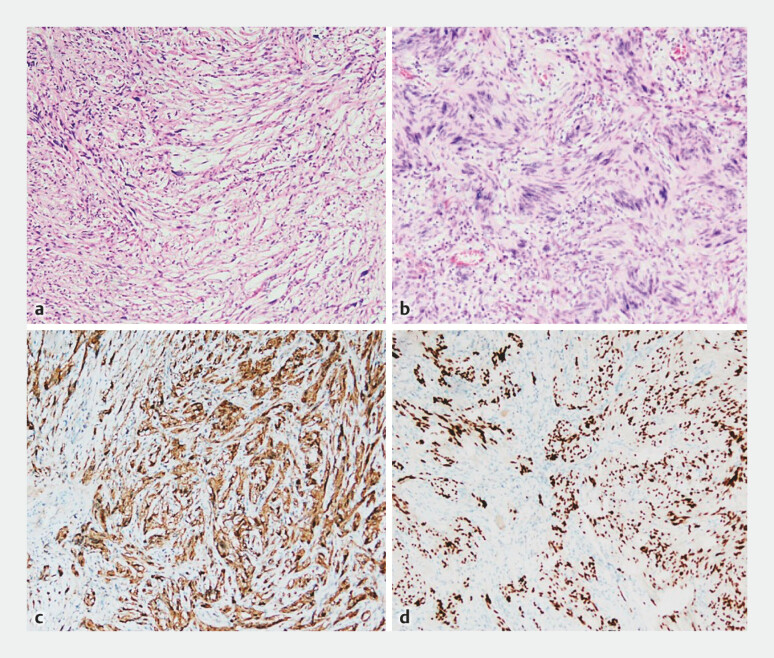
**a, b** Hematoxylin & eosin stain identified spindle cells. **c** Immunohistochemical expression of S100. **d** Expression of SOX 10.

While smaller muscular masses in the colon can be resected directly where perforations can be closed with clips or over-the-scope clips, the resection of larger masses poses significant challenges due to the difficulty of closing perforations 1 . Our experience confirms the feasibility of STER for colonic lesions, suggesting its potential for broader application in the future as technical proficiency improves and endoscopic equipment continues to evolve.

Endoscopy_UCTN_Code_TTT_1AQ_2AD_3AZ

## Notes

Endoscopy E-Videos https://eref.thieme.de/e-videos
*E-Videos* is an open access online section of the journal *Endoscopy* , reporting on interesting cases and new techniques in gastroenterological endoscopy. All papers include a high-quality video and are published with a Creative Commons CC-BY license. Endoscopy E-Videos qualify for HINARI discounts and waivers and eligibility is automatically checked during the submission process. We grant 100% waivers to articles whose corresponding authors are based in Group A countries and 50% waivers to those who are based in Group B countries as classified by Research4Life (see: https://www.research4life.org/access/eligibility/ ). This section has its own submission website at https://mc.manuscriptcentral.com/e-videos .
